# A Novel Cytochrome P450 2E1 Inhibitor Q11 Is Effective on Lung Cancer via Regulation of the Inflammatory Microenvironment

**DOI:** 10.1002/advs.202303975

**Published:** 2023-10-24

**Authors:** Lin Jia, Fei Gao, Guiming Hu, Yan Fang, Liming Tang, Qiang Wen, Na Gao, Haiwei Xu, Hailing Qiao

**Affiliations:** ^1^ Institute of Clinical Pharmacology Zhengzhou University Zhengzhou Henan 450001 China; ^2^ School of Pharmaceutical Sciences Zhengzhou University Zhengzhou Henan 450001 China

**Keywords:** CYP2E1, CYP2E1 Inhibitor, inflammation, lung cancer, tumor microenvironment

## Abstract

Lung cancer is the leading cause of death among all cancers. A persistent chronic inflammatory microenvironment is highly correlated with lung cancer. However, there are no anti‐inflammatory agents effective against lung cancer. Cytochrome P450 2E1 (CYP2E1) plays an important role in the inflammatory response. Here, it is found that CYP2E1 is significantly higher in the peritumoral tissue of non‐small cell lung cancer (NSCLC) patients and lung tumor growth is significantly impeded in *Cyp2e1^−/−^* mice. The novel CYP2E1 inhibitor Q11, 1‐(4‐methyl‐5‐thialzolyl) ethenone, is effective in the treatment of lung cancer in mice, which can inhibit cancer cells by changing macrophage polarization rather than directly act on the cancer cells. It is also clarify that the benefit of Q11 may associated with the IL‐6/STAT3 and MAPK/ERK pathways. The data demonstrate that CYP2E1 may be a novel inflammatory target and that Q11 is effective on lung cancer by regulation of the inflammatory microenvironment. These findings provide a molecular basis for targeting CYP2E1 and illustrate the potential druggability of the CYP2E1 inhibitor Q11.

## Graphical abstract

It is discovered and validated CYP2E1 as a novel inflammatory target in lung cancer by patients and *Cyp2e1^‐/‐^* mice. The novel developed CYP2E1 inhibitor Q11, 1‐(4‐methyl‐5‐thialzolyl) ethenone, is effective on lung cancer in mice via regulation of the macrophage polarization in the tumor inflammatory microenvironment.

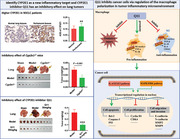

## 1 Introduction

Lung cancer starts in the lung, trachea, or bronchus,^[^ ^1^ ^]^ and has the highest morbidity and mortality rate among all cancers.^[^ ^2^, ^3^ ^]^ The cause of lung cancer is complex, and persistent chronic lung infections are one of the most important factors such as smoking, tuberculosis, and chronic obstructive pulmonary disease.^[^ ^4^ ^]^ Surgical resection is the main treatment for lung cancer, but it has a high recurrence rate and metastasis rate. Moreover, chemotherapy drugs and radiotherapy have only limited efficacy and strong toxicity in patients. In recent years, targeted drug therapy for lung cancer has made significant progress, such as mutant epidermal growth factor (EGFR) receptor inhibitors.^[^ ^5^ ^]^ However, this approach is limited to those who have the relevant mutations in their EGFR receptor. Thus, it is urgent to find new targets for the prevention and treatment of lung cancer.

Increasing evidence indicates that the tumor microenvironment plays an essential role during all stages of cancer progression.^[^ ^6^, ^7^, ^8^, ^9^ ^]^ Tumor‐associated inflammation is a key factor of the tumor microenvironment ^[^ ^9^, ^10^ ^]^ and plays a pivotal role in the occurrence and development of cancer,^[^ ^11^, ^12^, ^13^ ^]^ as well as participates in the various stages of tumor angiogenesis, proliferation, progression and metastasis.^[^ ^14^, ^15^ ^]^ Over the past few decades, the role of inflammation in cancer development, progression, and treatment has received much attention.^[^ ^16^ ^]^ The lung is an open organ exposed to the external environment, and is prone to external damage and induced inflammation.^[^ ^17^ ^]^ The persistent chronic inflammation caused by lung diseases such as tuberculosis is highly correlated with lung cancer.^[^ ^18^, ^19^, ^20^, ^21^ ^]^ These studies support the important role of inflammation in lung cancer, and, unfortunately, existing clinical anti‐inflammatory drugs such as nonsteroidal anti‐inflammatory drugs (NSAIDs) have no satisfied effect on lung cancer, which may be due to lack of novel effective inflammatory targets.

Cytochrome P450 2E1 (CYP2E1) is an important phase I metabolic enzyme, mainly found in the liver, but also found in lung, brain, kidney, and other organs.^[^ ^22^, ^23^ ^]^ CYP2E1 participates in the metabolism of various xenobiotics in the lung such as urethane, ethanol, and silica.^[^ ^24^ ^]^ During metabolism, ROS are produced and lead to oxidative responses and inflammation.^[^ ^25^ ^]^ Studies have shown that CYP2E1 is related to some inflammation‐associated pathways,^[^ ^26^, ^27^, ^28^, ^29^ ^]^ suggesting that CYP2E1 plays an important role in the inflammatory response. Furthermore, CYP2E1 has been reported to be closely related to pulmonary fibrosis, emphysema, and other lung diseases.^[^ ^30^ ^]^ Epidemiological studies show that a CYP2E1 gene polymorphism is associated with lung cancer susceptibility.^[^ ^31^ ^]^ Thus, it is worthy of further study whether CYP2E1 could be a potential inflammatory target in the lung tumor inflammatory microenvironment.

Although CYP2E1‐specific inhibitors have been available for many years,^[^ ^32^ ^]^ most of them lack inhibitory potency or have poor selectivity. Our previous study found that CYP2E1 could be a new anti‐inflammatory target in sepsis and glioblastoma.^[^ ^33^, ^34^ ^]^ We synthesized a novel CYP2E1 inhibitor, 1‐(4‐methyl‐5‐thialzolyl) ethenone, named Q11 with high selectivity and an inhibitory constant Ki for CYP2E1 of less than 1 µM. Q11 showed satisfactory bioavailability and pharmacokinetic characteristics after oral administration. Long‐term CYP2E1 inhibition by Q11 showed a good safety profile.^[^ ^33^ ^]^ And we found that Q11 is effective in sepsis mouse model and GBM mouse model via the anti‐inflammation effect.^[^ ^33^, ^34^ ^]^ In this study, we investigated the role of CYP2E1 in lung cancer, evaluated the effect of Q11 on lung cancer and identified the mechanism.

## 2 Results

### 2.1 Discovery and Validation of CYP2E1 as a Target in Lung Cancer

**Figure 1:**
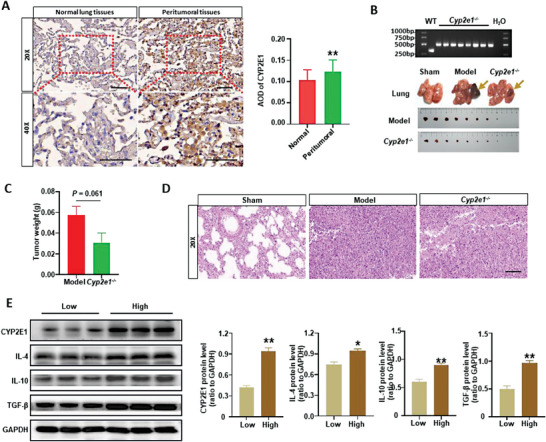
CYP2E1 is a potential target associated with inflammation in lung cancer. A) Representative IHC‐stained images for normal lung and peritumoral tissues of NSCLC patients, the average optical density between the two groups were compared, the number of samples in each group was 30, scale bar = 100 µm; B–D) The upper picture is the representative picture of *Cyp2e1^−/−^* mice genotyping by PCR, the band of WT mice is 317 bp, while the band of *Cyp2e1^−/−^* mice is 474 bp. The below picture is the representative picture of the lung with unstripped tumor tissues of mice, H&E staining was performed to identify the tumor and the tumor weight was evaluated, scale bar = 100 µm, Sham group (n = 5), Model group (n = 8), *Cyp2e1^−/−^* group (n = 8); E) The protein level of inflammatory cytokines IL‐4, IL‐10 and TGF‐β in peritumoral tissues of NSCLC patients was measured by western blot and quantified, the low group was CYP2E1 low expression group, and the high group was CYP2E1 high expression group. All data were presented as Mean ± SEM, *p< 0.05 or **p< 0.01 or ***p< 0.001.

To gain insight into how the expression of CYP2E1 was altered in lung cancer, we quantified the level of CYP2E1 in the peritumoral tissues of non‐small cell lung cancer (NSCLC) patients. We found that the CYP2E1 level was significantly higher in the peritumoral tissue of NSCLC patients than in normal lung tissue (**Figure** **1**A). To determine if CYP2E1 is a therapeutic target for lung cancer, we established the Lewis lung orthotopic xenograft model using *Cyp2e1* knockout (*Cyp2e1^−/−^*) mice, and H&E staining was performed to identify the tumor (Figure 1B–D). The results showed that *Cyp2e1* depletion had an obvious trend to inhibit the growth of lung tumors (Figure 1C, *P* = 0.061). Of note, we found greater levels of inflammatory factors such as IL‐4, IL‐10 and TGF‐β were associated with a higher CYP2E1 level in peritumoral tissues (Figure 1E), indicating that CYP2E1 may be related to the inflammation response in lung cancer. Together, these results demonstrate that CYP2E1 plays a critical role in lung cancer, and it may be a potential target associated with inflammation in lung cancer.

### 2.2 Q11 Shows Significant Anti‐Tumor Effects

**Figure 2:**
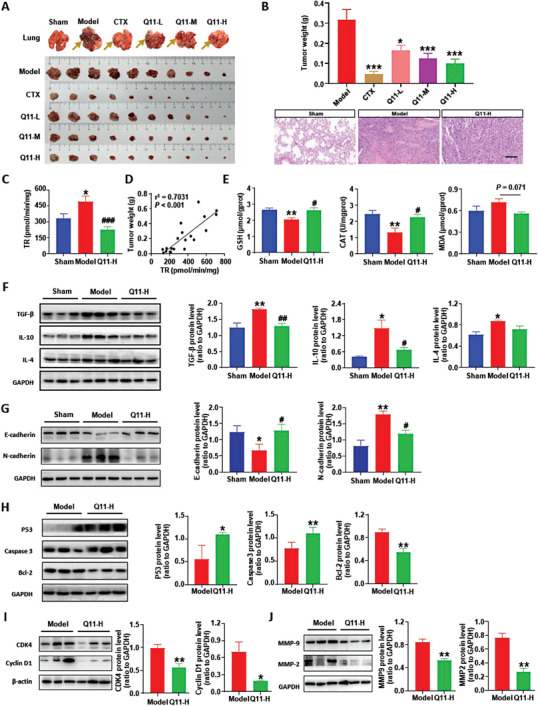
Q11 inhibits tumor growth by improving the tumor inflammatory microenvironment. A) The representative picture of the lung with unstripped tumor tissues of mice and the stripped tumor tissues were arranged from large to small; B) H&E staining was performed to identify the tumor and the total tumor weight were evaluated, scale bar = 100 µm, Sham group (n = 15), Model group (n = 20), CTX (20 mg k^−1^ g) group (n = 20), Q11‐L (3.3 mg k^−1^g) group (n = 15), Q11‐M (10 mg k^−1^g) group (n = 20), Q11‐H (30 mg k^−1^ g) group (n = 20); C) Q11 suppressed the elevation of CYP2E1 enzyme activity in mice induced by orthotopic xenograft of Lewis lung cells; D) The correlation between CYP2E1 activity and tumor weight in Lewis lung orthotopic xenograft mice model; E) The level of GSH, CAT and MDA in peritumoral tissues were evaluated; F) The protein level of inflammatory cytokines IL‐4, IL‐10 and TGF‐β in peritumoral tissues was measured by western blot and quantified; G) The protein level of EMT marker E‐cadherin and N‐cadherin in peritumoral tissues was measured by western blot and quantified; H) The protein level of P53, caspase3 and Bcl‐2 in tumor tissues was measured by western blot and quantified; I) The protein level of Cyclin D1 and CDK4 in tumor tissues was measured by westernblot and quantified; J) The protein level of MMP‐2 and MMP‐9 in tumor tissues was measured by western blot and quantified; In B and H‐J, * versus Model group; From C‐G: * versus Sham group, ^#^ versus Model group. All data were presented as Mean ± SEM, *p< 0.05 or **p< 0.01 or ***p< 0.001, ^#^ *p*< 0.05 or ^##^ *p*< 0.01 or ^###^ *p*< 0.001.

To investigate whether the CYP2E1 inhibitor is effective on lung cancer, we established the Lewis lung orthotopic xenograft model in WT mice with or without CYP2E1 inhibitor Q11 treatment. Cyclophosphamide (CTX) is a common antitumor drug, and we used CTX as a positive control drug. H&E staining was performed to identify the tumor, and we found that Q11 significantly inhibited the tumor growth in a dose‐dependent manner (**Figure** **2**A,B). Q11 was confirmed to significantly reduce the increase of CYP2E1 enzyme activity induced by orthotopic xenograft of Lewis lung cells in mice (Figure 2C). Notably, the correlation analysis showed that there was a positive correlation between CYP2E1 enzyme activity and tumor weight (Figure 2D, r^2^ = 0.7031, *P* < 0.001). These results suggest that Q11 is effective in lung cancer.

Next, we evaluated how Q11 inhibits tumor growth. Compared to the model group, the level of GSH and CAT was significantly increased in the Q11 treatment group (Figure 2E), indicating that Q11 could significantly reduce oxidative stress. Although there was no significant difference, the level of MDA showed a decreasing trend in the Q11 treatment group compared with the model group (Figure 2E), suggesting that Q11 may reduce the oxidative stress injury caused by lung tumors. Decreased levels of TGF‐β and IL‐10 in the Q11 treatment group indicated the anti‐inflammatory effect of Q11 (Figure 2F). In addition, we found that the level of E‐cadherin was significantly increased in the Q11 treatment group while the N‐cadherin was significantly decreased compared to the model group (Figure 2G), meaning that Q11 could restrain the EN switch. Increased P53 and caspase‐3 levels, and decreased Bcl‐2 (an anti‐apoptosis gene) level indicated that Q11 could promote cell apoptosis (Figure 2H). Decreased CDK4 and CyclinD1 levels, MMP‐9 and MMP2 levels indicated suppressed cell proliferation and cell invasion, respectively (Figure 2I,J). Thus, our results demonstrate that Q11 can improve the tumor microenvironment by inhibiting inflammatory responses and reducing oxidative stress damage, moreover, Q11 can promote cancer cell death and suppress cancer cell proliferation and invasion to prevent tumor growth, which may both contribute to the anti‐tumor effects of Q11.

### 2.3 Q11 Inhibits Cancer Cells through Macrophages

**Figure 3:**
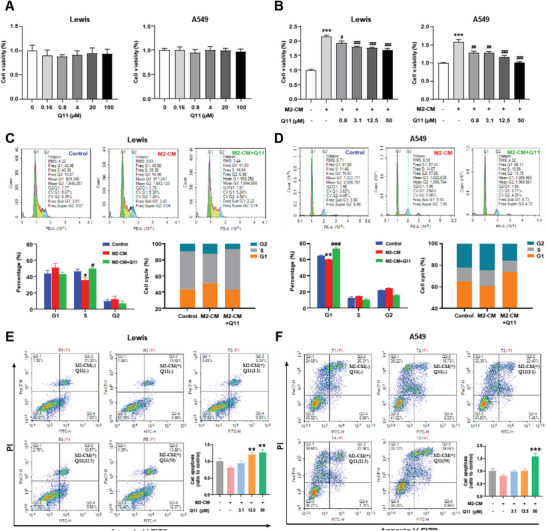
Q11 inhibits the proliferation and promotes the death of lung carcinoma cells. A) Cell viability of Lewis and A549 cells were determined by CCK8 assay; B) Cell viability of Lewis and A549 cells co‐cultured with M2‐CM were determined by CCK8 assay; C,D) Cell cycle of Lewis and A549 cells co‐cultured with RAW264.7 (C) and THP‐1 (D) M2‐CM, respectively, were determined by FACS and quantified; E,F) Cell apoptosis of Lewis and A549 cells co‐cultured with RAW264.7 (E) and THP‐1 (F) M2‐CM, respectively, were determined by FACS and quantified; In B: * versus M2‐CM(‐)Q11(‐) group, ^#^ versus M2‐CM(+)Q11(‐) group; In C and D: * versus control, ^#^ versus M2‐CM; In E and F: * versus M2‐CM(+)Q11(‐) group. All data were presented as Mean ± SEM, *p< 0.05 or **p< 0.01 or ***p< 0.001, ^#^ *p*< 0.05 or ^##^ *p*< 0.01 or ^###^ *p*< 0.001.

**Figure 4:**
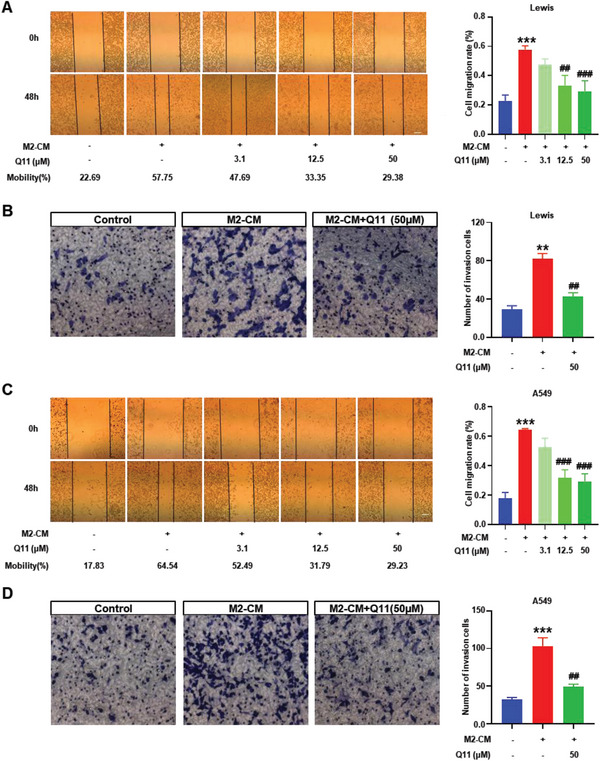
Q11 inhibits the migration and invasion of lung carcinoma cells. A) Cell migration of Lewis cells co‐cultured with RAW264.7 M2‐CM was detected by scratch assay, and cell migration rate was quantified; B) Cell invasion of Lewis cells co‐cultured with RAW264.7 M2‐CM was detected by transwell assay, and the number of invasion cells was quantified; C) Cell migration of A549 cells co‐cultured with THP‐1 M2‐CM were detected by scratch assay, and cell migration rate was quantified; D) Cell invasion of A549 cells co‐cultured with THP‐1 M2‐CM were detected by transwell assay, and the number of invasion cells was quantified. * Versus M2‐CM(‐)Q11(‐) group, ^#^ versus M2‐CM(+)Q11(‐) group. All data were presented as Mean ± SEM, *p< 0.05 or **p< 0.01 or ***p< 0.001, ^#^ *p*< 0.05 or ^##^ *p*< 0.01 or ^###^ *p*< 0.001.

To evaluate the effect of Q11 on cancer cells itself, we treated Lewis and A549 lung carcinoma cells with different concentrations of Q11. There was no inhibitory effect of Q11 on cell viability (**Figure** **3**A), indicating that the effect of Q11 may not be through direct inhibition of the cancer cells. Interestingly, Q11 significantly suppressed cell viability in a dose‐dependent manner when Lewis cells and A549 cells were co‐culture with M2 macrophages (Figure 3B). In addition, we found Q11 could induce cell cycle arrest at the S phase in Lewis cells (Figure 3C) and G1 phase in A549 cells (Figure 3D) in the co‐culture system with M2 macrophages, indicating that cancer cell proliferation was inhibited. Moreover, Q11 could significantly promote cell apoptosis in a dose‐dependent manner (Figure 3E,F). Then, we investigated the effect of Q11 on cancer cell migration and invasion. The scratch test showed that Q11 could obviously decrease the M2‐CM induced cell migration of Lewis and A549 lung cancer cells, especially in the Q11 50 µM group (**Figure** **4**A,C). The transwell assay revealed that Q11 could also significantly decrease M2‐CM induced cell invasion (Figure 4B,D). Together, these results indicate that Q11 cannot directly act on the cancer cells, but can inhibit their viability, migration and invasion through macrophages.

### 2.4 Q11 Changes Macrophage Polarization

**Figure 5:**
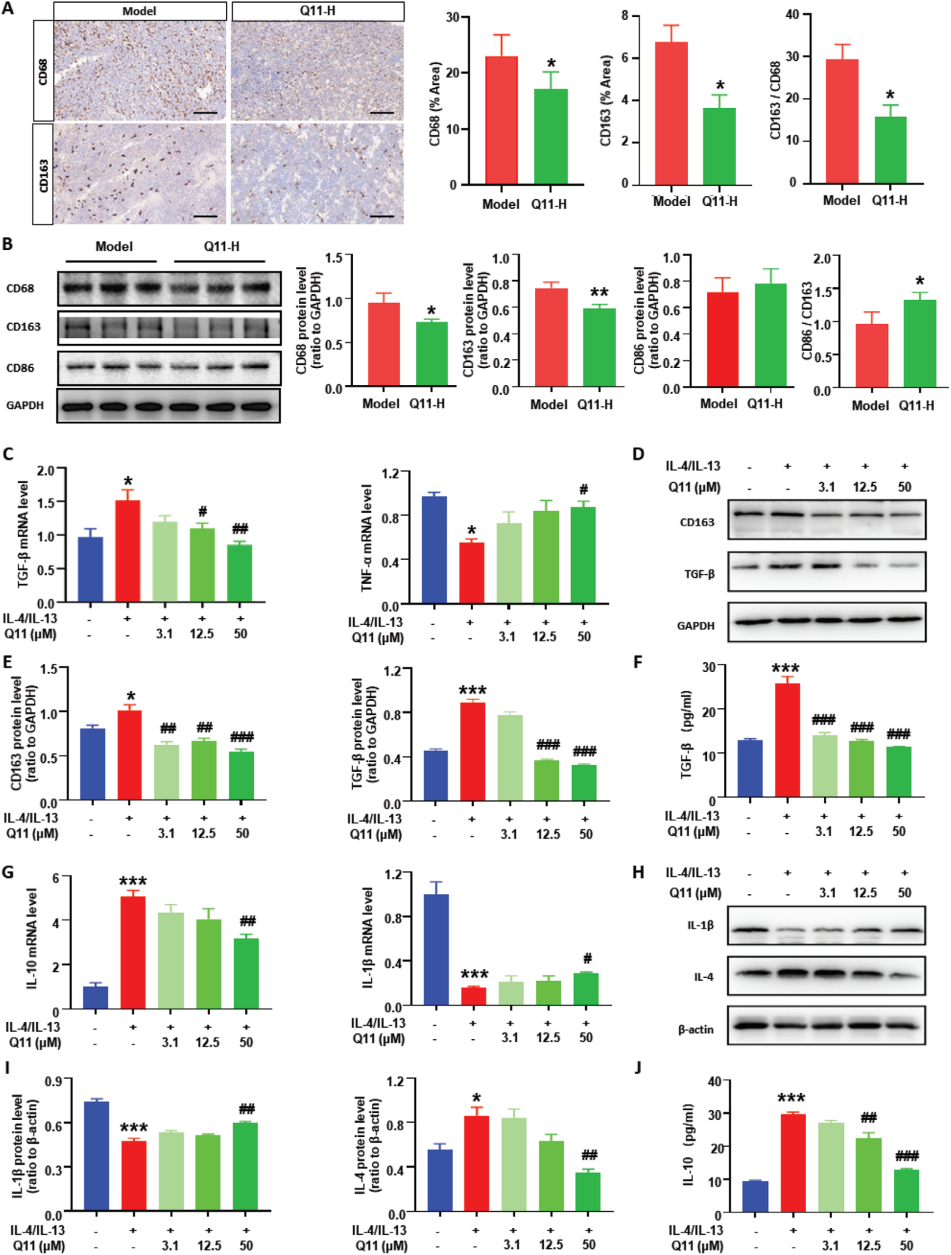
Q11 inhibits the M2 macrophage polarization but promotes M1 macrophage polarization. A) Immunohistochemical staining for CD68 (macrophage marker) and CD163 (M2 marker) on tumor tissue slices, the CD68‐positive and CD163‐positive area were quantified, scale bar = 100 µm, n = 6 for each group; B) The protein expression level of CD68, CD163 and CD86 in tumor tissues were measured by western blot and quantified; C) The mRNA levels of M2 marker TGF‐β and M1 marker TNF‐α in RAW264.7 cells treated with different concentration of Q11 were determined by RT‐PCR; D,E) The protein level of M2 markers CD163 and TGF in RAW264.7 cells treated with different concentration of Q11 was determined by western blot and quantified; F) Amount of IL‐10 in the THP‐1 cell supernatant was determined by ELISA; G) The mRNA levels of M2 marker IL‐10 and M1 marker IL‐1β in THP‐1 cells treated with different concentration of Q11 were determined by RT‐PCR; H,I) The protein level of M2 markers IL‐4 and M1 marker IL‐1β in THP‐1 cells treated with different concentration of Q11 was determined by western blot and quantified; J) Amount of TGF‐β in the RAW264.7 cell supernatant was determined by ELISA; The Q11‐H group represented in (A, B) was Q11 (30 mg k^−1^ g) group; From C‐J: * versus IL‐4/IL‐13(‐)Q11(‐) group, ^#^ versus IL‐4/IL‐13(+)Q11(‐) group. All data were presented as Mean ± SEM, *p< 0.05 or **p< 0.01 or ***p< 0.001, ^#^ *p*< 0.05 or ^##^ *p*< 0.01 or ^###^ *p*< 0.001.

To address how Q11 affects cancer cells through macrophages, we performed the immunohistochemical staining for CD68 (macrophage marker) and CD163 (M2 marker) on tumor tissue slices. We found that CD68‐positive and CD163‐positive immunoreactivity significantly decreased in the Q11 treatment group (**Figure** **5**A). Next, we measured the CD68, CD86 (M1 marker) and CD163 protein levels in tumor tissues. Compared to the model group, the protein level of CD68 and CD163 was significantly decreased in the Q11 treatment group (Figure 5B). Although the protein level of CD86 had no significant difference, the CD86 / CD163 ratio was significantly increased in the Q11 treatment group. Suggesting that Q11 could regulate macrophage polarization. We further measured the effects of Q11 on the polarization of RAW264.7 and THP‐1 macrophages in vitro. When we induced RAW264.7 cells into M2 macrophage by IL‐4 and IL‐13, the mRNA level of the M2 macrophage marker TGF‐β was significantly increased, while the M1 macrophage marker TNF‐α was significantly decreased compared to the IL‐4/IL‐13(‐)Q11(‐) group (Figure 5C). Interestingly, Q11 could significantly reverse the upregulated mRNA level of TGF‐β and the downregulated mRNA level of TNF‐α induced by IL‐4/IL‐13 in a dose‐dependent manner (Figure 5C). The TGF‐β protein level and another M2 marker, CD163, showed similar changes as the mRNA level (Figure 5D,E). We next determined the effect of Q11 on the secretion of tumor‐promoting inflammatory cytokine, and found that the TGF‐β level in the conditioned medium was significantly increased in the IL‐4/IL‐13 induced group but decreased upon Q11 treatment (Figure 5F). Consistent with the RAW264.7 results, the mRNA level of M2 macrophage marker IL‐10 was significantly increased while the M1 macrophage marker IL‐1β was significantly decreased in IL‐4/IL‐13‐induced group compared to the IL‐4/IL‐13(‐)Q11(‐) group in THP‐1 cells, and Q11 could significantly reverse the change in a dose‐dependent manner (Figure 5G). The protein level of the M2 macrophage marker IL‐4 was significantly increased and the M1 macrophage marker IL‐1β was significantly decreased in the IL‐4/IL‐13 induced group, but Q11 could significantly reverse this change (Figure 5H,I). The secretion of the tumor‐promoting inflammatory cytokine, IL‐10, was also significantly increased in the IL‐4/IL‐13 induced group, but decreased upon Q11 treatment (Figure 5J). Taken together, these data reveal that Q11 can inhibit M2 macrophage polarization and promote M1 macrophage polarization.

### 2.5 Q11 Inhibits the Activation of the IL‐6/STAT3 Pathway and the MAPK/ERK Pathway

**Figure 6:**
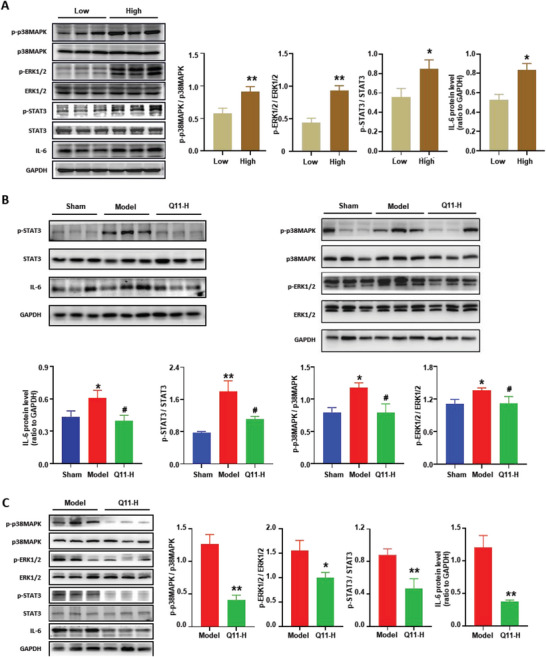
Q11 inhibits the activation of the IL‐6/STAT3 pathway and the MAPK/ERK pathway. A) The protein expression level of IL‐6, STAT3, p‐STAT3, ERK1/2, p‐ERK1/2, p38MAPK and p‐p38MAPK in peritumoral tissues of NSCLC patients were measured by western blot and quantified, the low group was CYP2E1 low expression group, and the high group was CYP2E1 high expression group; B) The protein expression level of IL‐6, STAT3, p‐STAT3, ERK1/2, p‐ERK1/2, p38MAPK and p‐p38MAPK in peritumoral tissues were measured by western blot and quantified; C) The protein expression level of IL‐6, STAT3, p‐STAT3, ERK1/2, p‐ERK1/2, p38MAPK and p‐p38MAPK in tumor tissues were measured by western blot and quantified; The Q11‐H group represented in (A, B) was Q11 (30 mg k^−1^ g) group; In A: * versus Sham group, ^#^ versus Model group. All data were presented as Mean ± SEM, *p< 0.05 or **p< 0.01 or ***p< 0.001, ^#^ *p*< 0.05 or ^##^ *p*< 0.01 or ^###^ *p*< 0.001.

To explore the potential mechanisms through which Q11 is effective in lung cancer, we examined the IL‐6/STAT3 pathway and the MAPK/ERK pathway (p38‐MAPK, ERK1/2), which were proven to play a critical role in tumorigenesis and the inflammatory response.^[^ ^35^, ^36^, ^37^ ^]^ We found that greater levels of IL‐6, phosphorylated STAT3, phosphorylated p38‐MAPK and phosphorylated ERK1/2 were associated with a higher CYP2E1 level in the peritumoral tissue of NSCLC patients (**Figure** **6**A). Compared to the sham group, the expression of IL‐6, phosphorylated STAT3 and two major MAPKs subtypes (phosphorylated p38‐MAPK and phosphorylated ERK1/2) were significantly increased in the lung cancer model group (Figure 6B). However, Q11 can markedly decrease cancer‐induced up‐regulation of those genes in peritumoral tissues (Figure 6B) and tumor tissues (Figure 6C). Together, these results revealed that Q11 can inhibit the activation of the IL‐6/STAT3 pathway and the MAPK/ERK pathway in lung cancer.

## 3 Discussion

Lung cancer has the highest morbidity and mortality rate among all cancers.^[^ ^2^ ^]^ Surgical resection and targeted therapy are common treatments used for lung cancer, but their efficacy is limited, and drug resistance is prone to occur. In recent years, the strategy for cancer treatment has turned to the tumor microenvironment.^[^ ^38^ ^]^ In the current study, we found that CYP2E1 could be a novel inflammatory target for the treatment of lung cancer, and our novel CYP2E1 inhibitor, Q11, is effective against lung cancer via regulation of inflammatory microenvironment.

Although studies have shown that CYP2E1 can participate in the occurrence and development of lung cancer and other lung diseases,^[^ ^30^, ^31^, ^39^ ^]^ the mechanism by which CYP2E1 affects lung cancer is unknown. For example, Ghanayem BI et al. found that the incidence of urethane‐induced lung cancer in *Cyp2e1^−/−^* mice is significantly reduced,^[^ ^39^ ^]^ however, their focus was on the contribution of CYP2E1 to urethane metabolism, not as a therapeutic target in lung cancer. In our study, we found that the level of CYP2E1 protein was significantly higher in the peritumoral tissues of NSCLC patients. We verified that *Cyp2e1* knockout can significantly inhibit tumor growth in a Lewis lung orthotopic xenograft model. In addition, we demonstrated a positive correlation between the CYP2E1 enzyme activity and tumor weight. Thus, our data reveal that CYP2E1 may be a potential target for prevention and treatment of lung cancer.

CYP2E1‐specific inhibitors have been available for many years,^[^ ^32^ ^]^ however, all of them have failed, perhaps due to insufficient inhibitory activity, poor selectivity and cytotoxicity. Our novel CYP2E1 inhibitor, Q11, specifically inhibits CYP2E1 enzyme activity with a high selectivity and high activity with an inhibitory constant Ki for CYP2E1 of less than 1 µM. In addition, the oral bioavailability and pharmacokinetic characteristics of Q11 are advantageous. Given the potential effects of CYP2E1 on lung cancer, we evaluated whether Q11 is effective on lung cancer. Our data show that Q11 has a good druggability in the treatment of lung cancer.

The tumor microenvironment, which is the environment around the tumor, includes fibroblasts, immune cells, inflammatory cells, stroma, microvessels, and extracellular matrix, biomolecules.^[^ ^8^ ^]^ Tumor‐associated inflammation is an important part of the tumor microenvironment, and has been considered as one of the key hallmarks of cancer, which will build a suitable environment for the tumor to grow by changing tissue homeostasis.^[^ ^11^, ^40^ ^]^ Our data show that Q11 can attenuate oxidative stress damage by improving the antioxidant capacity and decrease the inflammatory response. In addition, we found that Q11 can inhibit the activation of the IL‐6/STAT3 pathway and the MAPK/ERK pathways. Thus, our data demonstrate that Q11 can improve the tumor microenvironment by attenuating oxidative damage and inhibiting the inflammatory response, which may be associated with the IL‐6/STAT3 pathway and the MAPK/ERK pathway.

It has been reported that there are a large number of infiltrating inflammatory cells in the tumor stroma.^[^ ^41^ ^]^ Macrophages infiltrating in the tumor stroma are defined as tumor‐associated macrophages (TAMs), which are the most abundant infiltrating inflammatory cells in the tumor microenvironment.^[^ ^42^ ^]^ Although TAMs have two polarized phenotypes, M1 and M2, the M2‐like phenotype is more common in lung cancer.^[^ ^43^ ^]^ M1 macrophages exhibit an anti‐tumor phenotype, while M2 macrophages display a tumor‐promoting phenotype.^[^ ^44^, ^45^ ^]^ Intriguingly, we found that Q11 has no direct effects on lung cancer cells itself, but can inhibit lung cancer cell viability, migration and invasion by acting on macrophages. We further found that Q11 can inhibit M2 macrophage polarization and promote M1 macrophage polarization.

As mentioned above, although targeted drug therapy for lung cancer has made significant progress, its efficacy is limited. In our study, CYP2E1 was identified as a novel inflammatory target in lung cancer, which provides the possibility to develop new drugs for lung cancer. Q11, as a first‐in‐class CYP2E1 inhibitor, has obvious advantages in druggability and future clinical application. Drug resistance and toxicity have always been key problems in tumor therapy. Of note, our results show that Q11 can inhibit cancer cells by changing macrophage polarization rather than directly act on the cancer cells, this means that Q11 has no cytotoxic effect and cancer cells have difficulty to develop drug resistance. Furthermore, Q11 showed a good safety profile for 26 weeks in mice and 12 weeks in rats. Because many other tumors are also associated with tumor inflammatory microenvironments, and it is speculated that Q11 will be effective against many inflammation‐associated tumors, such as liver cancer, stomach cancer, colorectal cancer, etc. Thus, Q11 promises to be a good anti‐inflammatory agent for tumors of broad‐spectrum, low‐toxicity, and difficult‐resistance.

Our study has some limitations. The inflammation response and immune responses are closely linked and work together. Our results only demonstrate the effect of Q11 on regulating the tumor inflammatory microenvironment, however, the role of Q11 on immune responses also needs to be investigated. In addition, the combination of Q11 with other drugs targeting the tumor microenvironment needs further study, such as immunosuppressive drugs such as PD‐1/PD‐L1 antibodies and angiogenesis inhibitors such as bevacizumab, or chemotherapy drugs such as cyclophosphamide.

**Figure 7:**
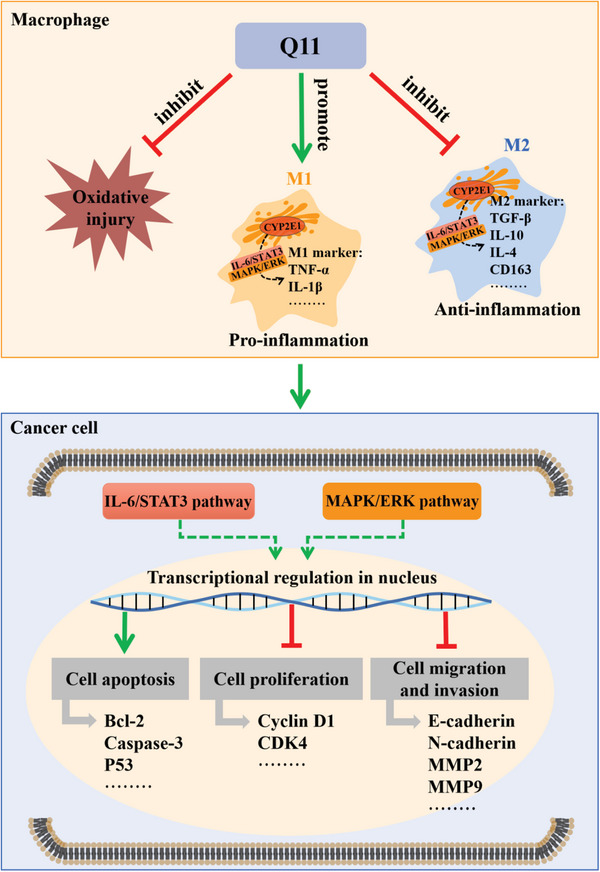
Schematic diagram of the CYP2E1 inhibitor Q11 in the inhibition of cancer cells via regulation of the macrophage polarization in tumor inflammatory microenvironment. Q11 inhibits M2 macrophage polarization, promotes M1 macrophage polarization and reduces oxidative injury. Moreover, Q11 cannot directly act on the cancer cells, but can inhibit their viability, migration and invasion by regulating macrophage polarization in the tumor inflammatory microenvironment, which may be associated with the IL‐6/STAT3 and MAPK/ERK pathway.

In summary, our study supports CYP2E1 as a new target in lung cancer. We demonstrate that a novel CYP2E1 inhibitor, Q11, is effective on lung cancer via regulation of the inflammatory microenvironment. In addition, we clarify that Q11 can inhibit the activation of the IL‐6/STAT3 pathway and the MAPK/ERK pathway, suggesting that Q11 regulates specific signaling pathways critical for inflammatory responses in lung cancer (**Figure** **7**). Our study provides new insight into the crucial role of CYP2E1 in lung cancer and demonstrates that CYP2E1 can be an inflammatory target in lung cancer. These findings provide a molecular basis for targeting CYP2E1 with small molecules, and illustrate the potential druggability of the CYP2E1 inhibitor Q11.

## 4 Experimental Section

### Specimen Collection

Normal lung samples (n = 30) and the peritumoral tissues of NSCLC patients (n = 30) were collected from Chinese patients who had undergone lung surgery at Second Affiliated Hospital of Zhengzhou University and Affiliated Cancer Hospital of Zhengzhou University. All protocols were approved by the Medical Ethics Committee of Zhengzhou University and conducted in accordance with the Declaration of Helsinki. The permit number for ethics approval was ZZUIRB2022‐152. All lung donors provided informed written consent.

### Mice

All animal experiments were approved by the Animal Ethics Committee of Zhengzhou University and were conducted in compliance with all relevant ethical regulations for animal testing and research. The permit number for ethics approval was ZZUIRB2022‐152. Mice were randomly selected for further biological analysis, and the investigators were blinded to group allocation during the experiments and outcome assessments.

### Cell Culture

Lewis and A549 lung carcinoma cells and RAW264.7 and THP‐1 macrophages cells were grown in Dulbecco's modified Eagle's medium (DMEM, Hyclone) supplemented with 10% fetal bovine serum (Gibco) and 1% penicillin‐streptomycin solution (100 units mL^−1^ penicillin, 100 µg mL^−1^ streptomycin; Invitrogen) in a humidified atmosphere containing 5% CO_2_ at 37 °C.

### Preparation of Liver Microsomes and Determination of CYP2E1 Enzyme Activation

Liver tissue was rinsed with 0.1 M Tris‐HCl buffer and surface moisture was absorbed with filter paper. Liver tissue (≈300 mg) was homogenized in three volumes of 2×PBS buffer. The prepared liver homogenate was centrifuged at 12 500×g for 20 min at 4 °C. The supernatant (800 µL) was mixed with CaCl_2_ (80 µL of 88 mM) and placed on ice for 5 min. Then the mixture was centrifuged at 12 500×g for 20 min at 4 °C and the supernatant was discarded. The pellet was washed with Tris‐HCl (0.1 M) buffer, and the pellet was resuspended with sucrose solution (0.25 M). The 100 µL microsomal incubation system contained diethylnitrosamine (160 mM) as substrate, with metabolites measured as described previously.^[^ ^46^ ^]^

### Lewis Lung Orthotopic Xenograft Mice Model

A small incision of 0.5‐1 cm was made at a distance of 1 cm from the scapula of the mouse and skin, and subcutaneous fat tissue was removed until the intercostal space and pleura could be clearly seen. Between the fourth rib and the fifth rib is the injection site. The Lewis cells suspension was mixed with an equal volume of Matrigel in 1 × 10^7^ mL^−1^ and 4 uL of the mixture (2 × 10^4^ cells inoculated) was slowly injected into the lung parenchyma using a microsyringe (placed on ice in advance). The sham group was injected with the same volume of DMEM basal medium. The incision was sutured layer by layer, and antibiotics were applied to prevent infection.

### Immunohistochemical Staining

The tissue sections were deparaffinized in xylene and rehydrated in a graded series of alcohols. Antigen retrieval was performed by steaming in distilled water for 30 min, and endogenous peroxidase activity was blocked by incubation in hydrogen peroxide (0.03%). Sections were then immunostained with anti‐CYP2E1 antibody (catalog no. ab28146, Abcam, 1:200), anti‐CD68 antibody (catalog no. GB113109, Servicebio, 1:200), anti‐CD163 antibody (catalog no. GB113751, Servicebio, 1:200). The stained slides were dehydrated, placed on a coverslip and scanned. The results were analyzed using Image Pro Plus 6.0.

### Oxidative Stress‐Marker Determination

The MDA, GSH and CAT levels were determined using a commercial kit according to the manufacturer's Instructions (catalog no. A006‐2‐1, A003‐1‐1, and A007‐1‐1 respectively, Nanjing Jiancheng Bioengineering Institute, China). Briefly, a 10% mouse lung tissue homogenate was prepared at a ratio of lung weight (mg): normal saline (µL) = 1:9 and centrifuged at 4500 r min^−1^ for 10 min. The protein concentration of the supernatant was determined by the BCA kit.

### The Indirect Co‐Culture System of Lung Cancer Cells and M2 Macrophages

THP‐1 monocytes were differentiated into macrophages with phorbol‐12‐myristate‐13‐acetate (PMA) (200 ng mL^−1^) for 24 h. THP‐1 macrophages and RAW264.7 macrophages were polarized into M2 type by incubation with 20 ng mL^−1^ of IL4 and IL‐13 for 48 h. The level of M1 and M2 markers were measured by RT‐PCR and western blotting to confirm the M2 status. The conditioned medium of THP‐1 M2 macrophages and RAW264.7 M2 macrophages were collected and co‐cultured with A549 lung cells and Lewis lung cells, respectively.

### Western Blot

All cells and tissues were lysed in RIPA buffer (Solarbio) and total protein concentrations were determined by BCA Protein Assay Kit (GLPBIO). Equal amounts of total protein (50 ug) were loaded for each sample and resolved by SDS–polyacrylamide gel electrophoresis and then transferred onto PVDF membranes (Millipore). Primary antibodies were incubated overnight at 4 °C and detection was performed using corresponding secondary antibodies by ECL western blot detection reagents (Millipore). The bands were quantified using ImageJ Software.

### RT‐PCR

Total RNA was isolated from tissues or cells using TRIzol reagent (Thermo Fisher Scientific). Reverse transcription was performed using ReverTra Ace qPCR RT Master Mix (Toyobo) and the resulting cDNA was used for qRT‐PCR. The set of β‐actin primers was used as an internal control for each specific gene amplification. The relative levels of expression were quantified and analyzed by using Bio‐Rad iCycleriQ software. The real‐time value for each sample was averaged and compared using the CT method, where the amount of target RNA (2^−ΔΔCT^) was normalized to the endogenous β‐actin reference (CT).

### CCK8

Cell viability was analyzed using the CCK8 assay. Briefly, cells were cultured in 96‐well plates at a concentration of 1.5 × 10^4^/well and treated with different concentrations of Q11 for 48 h. Then, CCK8 (10 µL) solution was added to each well and the cells were further incubated for 2 h. The absorbance was measured at 450 nm using an automated ELISA plate reader.

### ELISA

The IL‐10 and TGF‐β levels were measured using the commercial IL‐10 ELISA Kit (catalog no. EK110, Multi Sciences Biotech, Shanghai, China) and commercial TGF‐β ELISA Kit (catalog no. EK981, Multi Sciences Biotech, Shanghai, China) according to the manufacturer's instructions. Briefly, samples (100 µL) and detection antibodies (50 µL) were added to the plate and incubated for 2 hours at room temperature. After washing, the plate was incubated with HRP (100 µL) for 45 min at room temperature. After washing, the plate was then incubated with TMB (100 µL) for 5–30 min at room temperature. The reaction was stopped with Stop Solution (100 µL). The absorbance was measured at 450 and 630 nm using an automated ELISA plate reader.

### Transwell Assays

The cell migration assay was performed with transwell cell culture inserts comprised of two chambers separated by an 8.0 µm polycarbonate membrane (Costar, 3422). Cells (5 × 10^4^/well, 100 µL) suspended in serum‐free DMEM were added to the upper chamber of the inserts with serum‐free DMEM in the bottom chamber. After 30 min incubation at 37 °C, the bottom medium was replaced with DMEM containing 20% FBS. After 24 h the cells remaining on the upper surface of the membrane were removed by scraping with a cotton swab. Cells that migrated through the membrane were fixed with 4% paraformaldehyde, and then stained with 0.1% crystal violet solution (500 µL) for 10 min. The number of cells was counted with a Nikon inverted microscope.

### Statistical Analysis

All data are expressed as mean ± standard error (Mean ± SEM). GraphPad Prism 8.4.2 was used to plot the data, and SPSS 20.0 software was used for statistical analysis. For the data obeyed normal distribution, we used T‐test in two compared groups. If the data did not obey normal distribution, we used Mann‐Whitney U tests to perform non‐parametric T‐test in two compared groups. One‐way ANOVA was used to compare the means of multiple groups of data that obeyed normal distribution, and the Pearson test was used for correlation analysis. *P*< 0.05 was considered to have significant statistical difference, *p< 0.05 or **p< 0.01 or ***p< 0.001, ^#^ *p*< 0.05 or ^##^ *p*< 0.01 or ^###^ *p*< 0.001.

## Conflict of Interest

The authors declare no conflict of interest.

## Author Contributions

H.Q. performed conceptualization and experimental design, L.J., F.G., G.H., Y.F., L.T., Q.W., N.G., and H.X. performed experiments, L.J., F.G. performed data analysis, H.Q. performed funding acquisition, L.J. wrote the original draft, L.J. and H.Q. wrote, reviewed and edited.

## Data Availability

All the data in manuscript are available on request from the authors. The data and materials that support the findings of this study are available from the corresponding author, upon reasonable request.
